# Effects of non-invasive neurostimulation on autism spectrum disorder: A systematic review

**DOI:** 10.3389/fpsyt.2022.989905

**Published:** 2022-11-02

**Authors:** Jiawei Zhang, Hao Zhang

**Affiliations:** ^1^School of Rehabilitation, Capital Medical University, Beijing, China; ^2^Beijing Bo’ai Hospital, China Rehabilitation Research Center, Beijing, China; ^3^Cheeloo College of Medicine, Shandong University, Jinan, China; ^4^University of Health and Rehabilitation Sciences, Qingdao, China

**Keywords:** systematic review, meta-analysis, noninvasive neurostimulation, autism spectrum disorder, transcranial direct current stimulation, transcranial magnetic stimulation

## Abstract

Non-invasive neurostimulation techniques (NIBS) have shown benefits in psychiatric conditions. While in ASD patients, no guideline has so-far been recommended on these techniques due to a lack of high-quality synthetic evidence. Here, a comprehensive search from database inception onward was conducted in PubMed, EMBASE, and Cochrane library. Sham-controlled studies assessing the effects of NIBS in ASD patients were identified. After screening, twenty-two studies were included. A total of 552 patients were involved, and the sample size ranged from 5 to 78 patients. Although an iteration from exploratory attempts to more strictly designed trials has been seen to evaluate the efficacy of NIBS on ASD, further trials should also be needed to enable the clinicians and researchers to reach any consensus.

**Systematic review registration:**

[https://www.crd.york.ac.uk/prospero/display_record.php?ID=CRD42021292434], identifier [CRD42021292434].

## Introduction

Autism spectrum disorder (ASD) is a neurodevelopmental disorder marked by characteristic impairments in social communication and interaction, as well as restricted and repetitive behavior (1). According to the Diagnostic and Statistical Manual of Mental Disorders-Fifth Edition (DSM-V), social communication and social interaction consist of three aspects namely social-emotional reciprocity, non-verbal communicative behaviors used for social interaction, and developing, maintaining, and understanding relationships; and restricted, repetitive patterns of behavior displayed as stereotyped or repetitive movements, use of objects, or speech, insistence on sameness, unwavering adherence to routines, or ritualized patterns of verbal or non-verbal behavior, highly restricted, fixated interests that are abnormal in strength or focus, and increased or decreased response to sensory input or unusual interest in sensory aspects of the environment (2). The overall prevalence of ASD in Europe, Asia, and the United States has grown dramatically and was estimated to range from 2 to 25 per 1,000 (3). Having a child with ASD would cause considerable impacts to the family in various aspects including finances, physical and mental health of family members, and marital and sibling relationships (4). Regarding the economic burden of this condition, it requires as high as $2.4 million in the United States and £1.5 million in the United Kingdom to support an individual with an ASD and intellectual disability during his or her lifespan. While for an individual with an ASD without intellectual disability, the cost of was still high, estimated to be $1.4 million in the United States and £0.92 million (US $1.4 million) in the United Kingdom (5). To date, psychotherapy is the treatment of choice while only small-to-medium effects of improvement have been achieved (6). Therefore, there is a continued need for exploring effective interventions and evaluating treatment options for ASD.

In the past decade, non-invasive neurostimulation methods, including transcranial direct current stimulation (tDCS) and transcranial magnetic stimulation (TMS), have been proposed as potential therapeutic options for the modification of the pathological neuroplasticity (or even plasticity induction) involved in neuropsychiatric disorders including ASD (7, 8). Neuropathologic studies have demonstrated the presence of an excitation-inhibition imbalance within the cerebral cortex in both humans and animal models with ASD (9). NIBS either tDCS or TMS could induce current flow and neural activation in the targeted cortex when placed on the human scalp (10). The rationale to apply NIBS in dealing with ASD lies in the activation of certain inhibitory or excitatory neurons by NIBS could help restore its inherent balance of the cortex, thereby improving the corresponding function controlled by this cortex area (11). Nonetheless, different stimulation parameters and stimulation area of chosen may have caused considerably various effects on the patients.

The application of non-invasive neurostimulation on child and adult populations has proved to be well tolerated and showed a favorable therapeutic profile. However, no guideline has so-far been recommended on the application of NIBS in ASD due to a lack of high-quality synthetic evidence. A systematic review by Ali Khaleghi et al. has narratively synthesized published articles before April 2018 (12). It demonstrated that NIBS methods could be helpful for treating some dimensions of ASD such as repetitive behavior, sociability, or some aspects of executive and cognitive functions. However, a majority of studies included showed a moderate-to-low quality, and bias could easily be identified. Therefore, conclusion has been drawn to emphasize on further review and analysis on randomized, sham-controlled trials. Recently, trials investigating the effectiveness of NIBS on ASD patients are continuously rising. Christina Luckhardt et al. systematically searched for randomized, sham-controlled clinical trials of tDCS before May 2020 in individuals with ASD (13). Six eligible studies were identified, and the results indicated initial support for improved cognitive and social-communication skills in ASD following tDCS stimulation. García-González et al. also reported the results of their meta-analysis on the effects of tDCS on ASD (14). Three studies were included, and promising results for the use of tDCS were concluded. Nonetheless, the effects of TMS were not evaluated in their review. Therefore, a comprehensive review and evaluation of the up-to-date high-quality evidence in exploring effectiveness of NIBS including both tDCS and TMS in managing ASD patients are still warranted.

## Objectives

This study will systematically review available data on past, ongoing, and upcoming studies using TMS or tDCS as a therapeutic intervention in patients with ASD diagnosed by a solid method. This review will cover the following aspects:

1. Evaluating the effects of TMS and tDCS on core symptoms in patients with ASD.
2. Evaluating the effects of TMS and tDCS on neurocognition or psychiatric comorbidities other than core deficits.
3. Summarizing stimulation parameters that have been used in current TMS and tDCS administration and explaining how these parameters have affected the results.
4. Assessing the risk of bias and quality of the current evidence regarding this issue.

## Methods

The present protocol is being reported in accordance with the Preferred Reporting Items for Systematic Reviews and Meta-Analyses Protocols (PRISMA-P) statement (15). This protocol has been submitted within the International Prospective Register of Systematic Reviews (PROSPERO) database (PROSPERO, CRD42021292434). Any amendments made to this protocol when conducting the study will be outlined in PROSPERO and reported in the final manuscript. When this systematic review and meta-analysis being completed, it will be reported in accordance with the reporting guidance provided in the PRISMA statement (16). Since there is no human or animal experiment, the study will not require an ethics approval.

### Eligibility criteria

Studies will be included according to the following criteria: participants, interventions and comparators, outcome(s) of interest, and study design.

### Participants

Patients diagnosed with autism spectrum disorder based on a valid method will be included. The Diagnostic and Statistical Manual of Mental Disorders (DSM) is used predominantly in the United States (US) and has been updated to the fifth version (DSM-5) (2). The World Health Organization International Classification of Diseases, 10th revision (ICD-10) is used in other countries other than the United States (17). Other diagnostic tools include the Autism Diagnostic Interview-Revised (ADI-R) (18) and the Autism Diagnostic Observation Schedule (ADOS) (19).

### Interventions

Non-invasive neurostimulation including both transcranial direct current stimulation and transcranial magnetic stimulation will be included. Variation in tDCS like transcranial alternating current stimulation (tACS) will also be included. As for TMS studies, we will include low-frequency rTMS (LF-rTMS), high-frequency rTMS (HF-rTMS), intermittent theta burst stimulation (iTBS), continuous theta burst stimulation (cTBS), and paired associative stimulation (PAS), as well.

### Comparators

Sham stimulation: For tDCS, to make sure the participant was blinded to the procedure, the power indicator which was visible to the participants was lit up for both active and sham stimulation. However, in the sham stimulation condition, the current was discontinued normally after 30 s (20). For TMS, the sham stimulation was delivered with the coil tilted one-wing 90° off the head, which is a valid sham condition commonly used in double- or single-blind, sham-controlled trials (21).

### Outcomes of interest

Based on our preliminary searches, a variety of effect measurements have been used to evaluate the outcomes of non-invasive neurostimulation. These outcomes will be measured pre- and post-intervention. We will categorize these measurements into two major kinds, as test/scale/tasks and objective outcome measurements.

1. Test/scale/tasks includes but not limits to the Wisconsin Card Sorting Test (WCST), the Autism Spectrum Quotient (AQ), the Probabilistic Reversal Learning Task (PRLT), the Behaviour Rating Inventory of Executive Functioning (BRIEF; shift subscale), the Repetitive Behaviour Questionnaire 2A (RBQ-2A; total score), the Wechsler Memory Scale, 3rd Edition (WMS-III), the Brief Test of Attention (BTA), the Boston Naming Test (BNT), the Raven’s Progressive Matrices (RPM), the Movement Assessment Battery for Children-2 (MABC-2), the theory of mind test (TOMT), the featuring self-report clinical scales with good psychometric properties (RAADS), the Interpersonal Reactivity Index (IRI), the Childhood Autism Rating Scale (CARS), the Autism Treatment Evaluation Checklist (ATEC), the Clinical Global Impression-Improvement (CGI-I), the Aberrant Behavior Checklist (ABC), the Social Responsiveness Scale (SRS), the Repetitive Behavior Scale—Revised (RBS), the Eye-tracking apparatus, the Conner’s Continuous Performance Test (CCPT), the Yale-Brown Obsessive Compulsive Scale (Y-BOCS), the Frith–Happe animations task, the Reading the Mind in the Eyes test (RMET), the Global Clinical Impression Scale (GCIS), the Autism Diagnostic Interview, Revisited Edition (ADI-R), the 3-back task, Empathy Quotient (EQ), the facial emotion recognition and processing (FERP) test, the verbal fluency (VF) test, the Test of Adolescent Social Skills-Modified (TASSK-M), Children’s Sleep Habits Questionnaire (CSHQ), the Cambridge Neuropsychological Test Automated Battery (CANTAB), and the Mini International Neuropsychiatric Interview (MINI).

2. Objective outcome measurements include the metabolite levels, the functional magnetic resonance imaging (fMRI), and electroencephalogram (EEG).

### Study design

We will consider randomized controlled trials, non-randomized controlled trials, open-label trials, and crossover trials. Cohort, case control, case series, and case reports will also be searched for but not be included in the final manuscript. Only studies published in English will be included.

### Information sources and search strategy

PubMed, Embase (1996–2021 Week 43), and Cochrane library databases will be retrieved according to the search strategy. No limitations regarding the study design, publication time, and age or sex of participants will be set when searching for the records. A draft search strategy is present in Supplementary Table 1.

### Study selection

Studies will be selected based on the following *inclusion criteria*: (1) Sham-controlled designed studies comparing the effects of any non-invasive neurostimulation with that of a sham stimulation group; (2) studies enrolling patients with solid diagnosis of autism spectrum disorder; (3) studies providing outcomes measuring the therapeutic or side effects. *Exclusion criteria* will be (1) studies reported in language other than English; (2) studies published in the form of editorial, comments, or conference abstract, in which details of PICOS were not reported; (3) studies comparing the responses of patients with ASD to non-invasive neurostimulation with that of other human subjects.

Records from database searches will be exported into EndNote and duplicates removed. Two reviewers will independently screen titles and abstracts for potentially eligible studies. The reviewers will then independently screen the full text of potentially eligible studies. Reasons for exclusions will be reported. At each stage of the review process, disagreements will be resolved by discussion or, if not achieve consensus, consulting a third review author acted as an arbitrator. The reviewers were not blinded to names of authors, institutions, outcomes, or journals.

### Data extraction

Two authors will extract data independently from the included articles, using a pre-developed form adapted from the Cochrane data collection form for intervention reviews and extracted data form published by Ulrike Schmidt et al. (22, 23).

#### General information

First author of study, published date, published journal, DOI, and author’s contact information (if available).

#### Study details

Study design; country; setting; sample size; inclusion and exclusion criteria; comparability of groups; study period; stratification; stopping rules; funding source; and conflict of interest.

#### “Risk-of-bias” assessment and justification for this judgment

Sequence generation; allocation concealment; blinding (participants, personnel, outcome assessors); incomplete outcome data; selective outcome reporting; and other bias (recall bias). Characteristics of participants: age; gender; ethnicity; the number randomized, analyzed and lost to follow-up; and dropouts in each arm (with reasons).

#### Interventions

Experimental and control interventions; details of non-invasive stimulation procedures (for current stimulation: anode site, cathode site, current, electrode size; for magnetic stimulation: coil placement, frequency, motor threshold, pulses); timing of intervention; and uptake of intervention (acceptance of stimulation), whether studies assessed adherence (compliance) with interventions.

#### Outcomes measured

Tests, scales, tasks, EEG, and metabolite measurement.

### Risk of bias in individual studies

We will use the revised Cochrane risk-of-bias 2 (RoB-2) tool to assess the quality of each trial (24). Two reviewers will independently score each trial, and each quality item will be graded as low risk, high risk, or unclear risk. We will resolve any discrepancies by consulting a third review author. We will assess the risk of bias for the seven domains: randomization sequence generation (selection bias), allocation concealment (selection bias), blinding of participants and personnel (performance bias), blinding of outcome assessment (detection bias), incomplete outcome data (attrition bias), selective reporting, and other bias. The included trials will be graded as low quality, moderate quality, or high quality based on the evaluation. To evaluate the possibility of publication bias, we will use funnel plots for analyses that contained more than 10 studies.

### Data synthesis

We will perform a narrative synthesis around the features of existing evidence investigating the effects of non-invasive neurostimulation on ASD patients. All eligible trials will be summarized in narrative form. Tables will be constructed based on the information extracted (Table 1). These tables will include key study characteristics such as study design, population, diagnosis tool, randomization methods, intervention parameters, sham stimulation methods, and outcomes.

**TABLE 1:** Characteristics of studies on sham-controlled clinical trials on transcranial direct current stimulation/transcranial magnetic stimulation interventions in autism spectrum disorder.

Study	Design			Diagnosis	NIBS technique	Outcome measures		
	Randomization	Blinding	Control			Test/scale/ task	Objective outcomes	Results
Ni et al. (29)	Yes, cross-over	Single blind	Sham	DSM-IV	rTMS (iTBS)	WCST, AQ		Positive
Parmar et al. (31)	Yes, cross-over	Double blind	Sham	DSM-V	tDCS	PRLT, BRIEF, RBQ-2A	EEG	Negative
Desarkar et al. (35)	Yes, cross-over	Open-label	Sham	RAADS-R	rTMS (TBS)		EEG	Positive
Van Steenburgh et al. (36)	Yes, Cross-over	Single blind	Sham	ADOS	tDCS	WMS-III, BTA		Positive
Mahmoodifar and Sotoodeh, (41)	Yes	Not mentioned	Sham	DSM-IV	tDCS	RPM, MABC-2		Positive
Salehinejad et al. (37)	Yes, Cross-over	Double blind	Sham	DSM-V, GARS	tDCS	TOMT		Positive
Enticott et al. (21)	Yes	Double blind	Sham	DSM-IV	rTMS	RAADS, AQ, IRI		Positive
Amatachaya et al. (42)	Yes, Cross-over	Double blind	Sham	DSM-IV TR	tDCS	CARS, ATEC, CGI-I		Positive
Qiao et al. (38)	Yes	Single blind	Sham	AQ-Chinese	High-definition tDCS	Eye-tracking apparatus, Free-viewing task		Positive
Ni et al. (43)	Yes, cross-over	Not mentioned	Sham	DSM-IV, ADI-R Chinese, ADOS	rTMS (iTBS)	CCPT, WCST: CV4, Y-BOCS, SRS		Positive
Liu et al. (27)	Yes	Double blind	Sham	AQ	rTMS (iTBS)	Eye-tracking apparatus	FMRI	Positive
Ni et al. (30)	Yes	Single blind	Sham	DSM-IV/DSM-V	rTMS (iTBS)	SRS, Frith–Happe animations task, RMET, RBS-R		Negative
Moxon-Emre et al. (28)	Yes	Double blind	Sham	DSM-IV-TR/PDD-NOS/DSM-V	rTMS		GlX and GABA	Positive
Amatachaya et al. (33)	Yes, cross-over	Double blind	Sham	DSM-IV TR	tDCS	ATEC	EEG	Positive
Fujino et al. (39)	No, cross-over	Single blind	Sham	DSM-IV-TR	rTMS (iTBS and cTBS)		FMRI	Positive
Abdullah habib et al. (53)	Yes	Single blind	Sham	ADOS	tDCS	3-back task		Positive
Hadoush et al. (40)	Yes	No	Sham	Not mentioned	tDCS	ATEC		Positive
Wilson et al. (34)	Yes, cross-over	Double blind	Sham	AQ	tDCS	EQ, FERP		Positive
Esse Wilson et al. (44)	Yes, cross-over	Double blind	Sham	AQ	tDCS	VF test, TASSK-M		Positive
Qiu et al. (20)	Yes	Single blind	Sham	DSM-V	tDCS	CARS, ABC, RBS-R, CSHQ		Positive
Zhou et al. (45)	Not mentioned	Double blind	Sham	PEP-III/DSM-IV-TR	tDCS		EEG	Positive
Ameis et al. (26)	Yes	Double blind	Sham	DSM-IV/DSM-V/ADOS-2	rTMS	VABS-II, MINI, CANTAB-SWM, BRIEF-MCI, A, T, SR		Negative

There may be a chance for meta-analysis to pool the estimates of studies included if three studies or more meet the requirements for meta-analysis. If so, a random-effect model maybe applied. We will evaluate heterogeneity between studies using the *I*^2^ statistic. We planned not to pool data where there was considerable heterogeneity (*I*^2^ ≥ 75%) that could not be explained by the diversity of methodological or clinical features among studies.

### Subgroup analyses

If there are sufficient data provided by the qualified studies, we will do meta-analyses stratified by age; sex; stimulation site, randomization procedures, stimulation intensity, montage, duration, and number of stimulation session. We will use the test for subgroup differences available in RevMan 5 to determine whether there was evidence for a difference in treatment effect between subgroups.

### Sensitivity analyses

Potential reasons for heterogeneity will be explored in sensitivity analyses; the pre-specified subgroup analyses, if feasible, will be examined to determine potential reasons for any observed statistical heterogeneity.

### Strength of evidence

The overall quality of evidence for all outcomes will be evaluated using the Grading, Recommendations, Assessment, Development and Evaluation (GRADE) framework, estimating individual risk of bias, meta-bias, precision, consistency, directedness, and the magnitude of effect (25). These indicators will determine the certainty of the estimated effect, which will be rated as either very low, low, high, or very high.

## Results

### Search results

**FIGURE 1:**
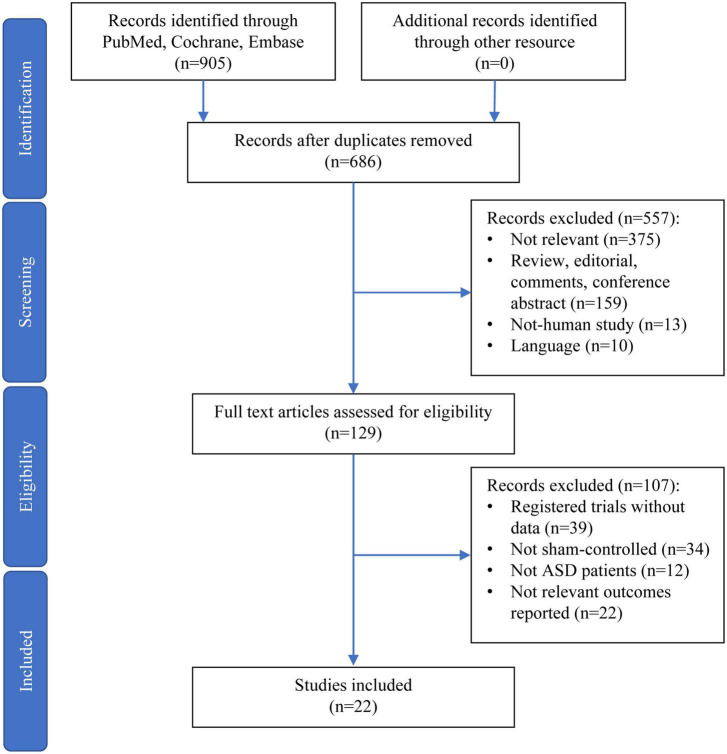
Preferred Reporting Items for Systematic Reviews and Meta-Analyses diagram for the selection of papers.

A total of 905 records were retrieved through electronic and manual searches. After 219 duplicates were removed, the titles and abstracts of 686 records were further screened. Among these records, 375 were not relevant to the objectives of this study, 159 were either reviews, editorials, comments, or conference abstracts, 13 were non-human study, and 10 were not reported in English. These records were excluded, therefore leaving 129 records for a full-text assessment. Finally, 22 articles were included and analyzed in this study after excluding those 107 records for not fulfilling the inclusion criteria (Figure 1).

### Characteristics of included trials

Twenty-two studies included were all sham-controlled studies (Table 1). These studies were published from 2011 to 2021, with 15 (65.2%) published after 2020. Of these studies, 20 were randomized designed, 1 was non-randomized, and 1 was not mentioned at all. The double blinding strategy was applied in 11 studies, while the single blinding strategy was in seven studies, open-labeled in two studies, and not mentioned in two studies. A total of 552 patients were involved, and the sample size ranged from 5 to 78 patients with a median of 20. The mean age ranged from 6.4 to 32.1 years old. Of note, 15 studies recruited ASD patients with an average age above 18 years old, while the left seven studies included those aged below 18. Most included patients were diagnosed by DSM-IV or DSM-V. Tools such as the Ritvo Autism Asperger Diagnostic Scale-Revised (RAADS-R), ADOS, and the Autism Spectrum Quotient (AQ) (or AQ Chinese version) were also applied. Among included studies, 13 investigated the effects of tDCS on ASD patients while nine focused on the effects of TMS. Three studies reported no significant effects of NIBS on ASD, while seven studies reported positive effects. Nonetheless, there is a very large heterogeneity in the outcomes reported in the included studies, as shown in Table 1. A wide range of tools were applied in assessing the effects of non-invasive neurostimulation on ASD, which were discussed in detail in the following section of primary analysis.

### Risk-of-bias assessment

**FIGURE 2:**
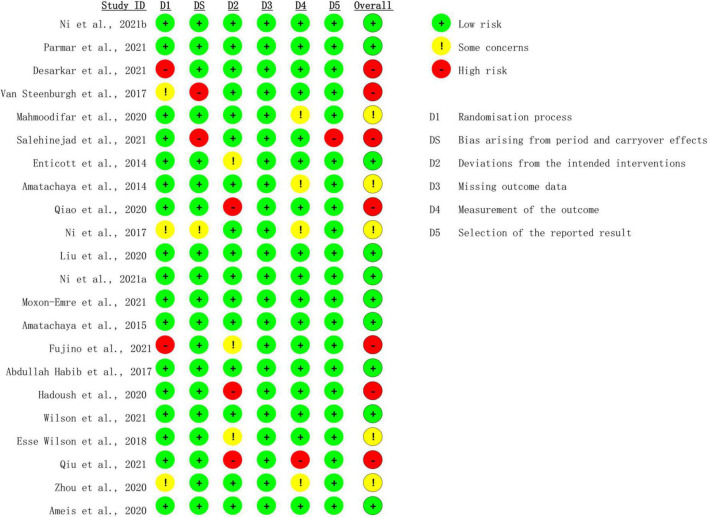
Summary of risk-of-bias analysis.

Risk of bias was assessed with the Cochrane risk-of-bias 2 (ROB-2) tool (24). There were 10 studies rated as low risk of bias (21, 26–34), whereas seven studies were rated as high risk of bias (20, 35–40), and the rest five studies were rated as moderate risk of bias (41–45) (Figure 2). Two studies showed high risk regarding the randomization process (35, 39), among which one study used open-label design, and the other was non-randomized. Bias arising from period and carryover effects was seen in two trials (36, 37). Three trials demonstrated deviation from the intended interventions (20, 38, 40). The measurement of the outcome was inadequately reported in one trial (20), and the selection of the reported results was shown in one trial (37).

### Narrative synthesis around features of the trials

We first synthesized the trials rated as low risk of bias were then considered for the synthesis. Among these trials, four investigated the efficacy of tDCS on ASD while six focused on TMS. However, diversity remains among these trials regarding the stimulation parameters and outcome measurements (Table 2). For those four tDCS studies, one study placed anode site on the right ventrolateral prefrontal cortex (vlPFC) (31), two on the left dorsolateral prefrontal cortex (DLPFC) (32, 33), and one on the right temporoparietal junction (rTPJ) (34). The current varying from 1 to 2 mA, the stimulation duration ranging from 15 to 30 min, and the total number of stimulation session around 2–5 times were applied by these trials. Unfortunately, the outcome measurement varied among these studies. Parmar et al. applied the Probabilistic Reversal Learning Task (PRLT), Behaviour Rating Inventory of Executive Functioning (BRIEF; shift subscale), and Repetitive Behaviour Questionnaire 2A (RBQ-2A; total score) to assess the cognitive response to the stimulation (31). Wilson et al. administrated Empathy Quotient (EQ) and a facial emotion recognition and processing (FERP) test to evaluate the outcomes (34). In addition, the autism treatment evaluation checklist (ATEC) and 3-back task were utilized by Amatachaya et al. (33) and Abdullah Habib (32), respectively. With respect to TMS trials, three trials stimulated posterior superior temporal sulcus (pSTS) with one unilaterally (27) and two bilaterally (29, 30). Two trials chose to intervene over bilateral DLPFC (26, 28), and one focused on bilateral dorsomedial prefrontal cortex (dmPFC) (21) (Table 3). Similarly, TMS at 50 Hz with 600 pulses in total, namely intermittent theta burst stimulation (iTBS), was given to the participants in those trials focused on pSTS (29, 30). Nonetheless, the number of sessions varied. For DLPFC, researchers applied TMS at 20 Hz with a total of 1500 pulses for 20 sessions to the ASD subjects. Enticott et al. who targeted dmPFC used a lower frequency of TMS at 5 Hz (21). One thousand and five hundred pulses per session for a total of 10 sessions of TMS were then tested. Outcome measurement diversity was also observed in these trials. Only Ni et al. and Enticott et al. both administrated Autism Spectrum Quotient (AQ) as one of their outcome measurements (21, 29). In addition, Ni et al. also used the Wisconsin Card Sorting Test (WCST) for cognitive flexibility measurement while Enticott et al. added Ritvo Autism-Aspergers Diagnostic Scale (RAADS) and Interpersonal Reactivity Index (IRI) to their outcome evaluation (21, 29). In the other study by Ni et al., they applied the Reading the Mind in the Eyes test (RMET) and Frith–Happe animations task. Given these two tasks, nonetheless, are not specific to the pSTS activities, they also adopted the repetitive behavior scale-revised (RBS-R) as the exploratory outcome (30). Interestingly, both Liu et al. and Moxon-Emre et al. showed interest in the objective outcome, thereby fMRI and GlX and GABA level was applied for outcome assessment (27, 28). A different set of scales including BRIEF-MCI and the Cambridge Neuropsychological Test Automated Battery (CANTAB) task were adopted by Ameis et al. (26).

**TABLE 2:** Stimulation parameters for transcranial direct current stimulation.

Study	DCS				Duration	Montage	Number of session
	Anode site	Cathode site	Current (mA)	Electrode size (cm^2^)			
Parmar et al. (31)	Right vlPFC	–	1.693	NR	20 min	Unilateral	4
Van Steenburgh et al. (36)	DLPFC	DLPFC	1.5	25	40 min	Bilateral	3
Mahmoodifar and Sotoodeh, (41)	left M1	Right supraorbital region	1.5	35	20 min	Unilateral	10
Salehinejad et al. (37)	vmPFC, Right TPJ	Left shoulder	1	25	20 min	Unilateral	3
Amatachaya et al. (42)	Left DLPFC	Right shoulder	1	35	20 min	Unilateral	5
Qiao et al. (38)	Right TPJ	C4, P4, T8, and P8	2	NR	20 min	Unilateral	5
Amatachaya et al. (33)	Left DLPFC	Right shoulder	1	35	20 min	Unilateral	1
Abdullah Habib et al. (53)	DLPFC	Contralateral supraorbital area	1.5	35	15 min	Bilateral	3
Hadoush et al. (40)	Prefrontal and motor areas	Contralateral supraorbital areas	1	8	25 min	Bilateral	10
Wilson et al. (34)	Right TPJ	Ipsilateral deltoid	2	11	30 min	Unilateral	2
Esse Wilson et al. (44)	Right TPJ	Ipsilateral deltoid	2	11	30 min	Unilateral	2
Qiu et al. (20)	Left DLPFC	Right shoulder	1	25	20 min	Unilateral	1
Zhou et al. (45)	Left DLPFC	Right eyebrow	1	30	20 min	Unilateral	1

NR, not report.

**TABLE 3:** Stimulation parameters for transcranial magnetic stimulation (TMS).

Study	TMS				Montage	Number of sessions
	Coil placement	Frequency (Hz)	MT (%)	Pulses		
Ni et al. (29)	pSTS	50	80 vs 60	600	Bilateral	5
Desarkar et al. (35)	Left M1	20	90	6000	Unilateral	1
Enticott et al. (21)	dmPFC	5	50	1500	Bilateral	10
Ni et al. (43)	DLPFC, pSTS	50	80 vs 60	600	Bilateral	4
Liu et al. (27)	Right pSTS	50	70	600	Unilateral	3
Ni et al. (30)	pSTS	50	80 vs 60	600	Bilateral	8
Moxon-Emre et al. (28)	DLPFC	20	90	1500	Bilateral	20
Fujino et al. (39)	Right TPJ	50	Not report	600	Unilateral	1
Ameis et al. (26)	DLPFC	20	90	1500	Bilateral	20

NR, not report.

Among the seven studies which were rated as high risk of bias, two reported on TMS and five on tDCS. Desarkar et al. place the TMS coil over left M1 area using a frequency at 20 Hz, while Fujino et al. stimulated the rTPJ with 50 Hz TMS (35, 39). Both trials evaluated the effects of one-session TMS stimulation based on objective measurements, namely EEG and fMRI. With respect to the five tDCS trials with high risk of bias, Van Steenburgh et al. (36) and Qiu et al. (20) placed the anode site on DLPFC, Salehinejad et al. (37) on rTPJ and vmPFC, Qiao et al. (38) on rTPJ, and Hadoush et al. (40) on prefrontal and motor areas. Qiao et al. (38) applied 2 mA current, Van Steenburgh et al. (36) used 1.5 mA, and the rest three trials administered 1 mA to the participants. Ten sessions of stimulation were performed by Hadoush et al. (40), five by Qiao et al. (38), three by Van Steenburgh et al. (36) and Salehinejad et al. (37), and one by Qiu et al. (20) All these five tDCS trials reported their findings using different outcome measurements, for example, WMS-II, TOMT, ATEC, and CARS.

The rest five studies with moderate risk of bias include one TMS trial and four tDCS trials. Ni et al. (30) stimulated bilateral DLPFC and pSTS with TMS at 50 Hz with 600 pulses for a total of four sessions. The outcome was measured by using scales namely CCPT, WCST: CV4, Y-BOCS, and SRS. Regarding the tDCS trials, two trials focused on left DLPFC (42, 45), one on left M1 area (41), and one on rTPJ (44). Outcome measurement heterogeneity was also observed in these trials. Zhou et al. (45) only used EEG to evaluate the effects of sDCS, and the rest four trials used various measurements such as RPM, CARS, and TASSK-M.

### Outcome synthesis of transcranial direct current stimulation trials

Parmar et al. conducted a pilot study comparing the effects of active anodal high-definition transcranial direct current stimulation (aHD-tDCS) with sham stimulation over the right vlPFC in ASD adolescents and young adults (31). The outcomes were measured by RN, PRLT, BRIEF-A, and RBQ-2A to evaluate cognitive flexibility before and after stimulation. However, improvements in cognitive flexibility following tDCS stimulation were not observed. In the study by Wilson et al., verum or sham tDCS was randomly assigned to recruited adults with ASD but no intellectual disability (34). The outcomes were measured using scores from the EQ and a FERP test. As concluded in their study, tDCS over rTPJ significantly improved EQ scores and FERP scores for emotions that conveyed threat. Nonetheless, in their designed, patients also received FERP, emotion, and empathy treatment interventions pairing with either verum or sham tDCS. Amatachaya et al. randomly assigned twenty male children with ASD to receive a single session of both active and sham tDCS stimulation (11 mA) over DLPFC (33). A crossover design was utilized in this study. The results show considerable improvements in social and health/behavior domains of ATEC. Moreover, they evaluated the EEG alteration and found that the peak alpha frequency (PAF) also increased at the stimulation site and associated with the improvements in the two domains of ATEC. For the last tDCS trial with low risk of bias, Abdullah Habib et al. focused on the working memory impairments in individuals with ASD (32). Twenty-five adults with high-functioning autism (HFA) (Mean age: 25.81, range 18–35) were recruited and randomized to tDCS or sham stimulation over DLPFC. The 3-back task was applied to evaluate the efficacy of intervention on working memory. The trial revealed that anodal tDCS administered over the left DLPFC enhanced WM in terms of the recognition accuracy in individuals with HFA. Interestingly, all the trials with moderate and high risk of bias reported significant therapeutic effects of tDCS on ASD, despite various stimulation parameters and outcome measurements they applied.

### Outcome synthesis of transcranial magnetic stimulation trials

Enticott et al. performed deep rTMS over bilateral dorsomedial prefrontal cortex to 28 adults with ASD (21). They found that active deep rTMS significantly reduced social relating impairments as measured by the RAADS and decreased self-oriented anxiety in difficult social environments as measured by the IRI, as compared to sham stimulation. Two trials by Ni et al. were included for analysis (29, 30). They were interested in the efficacy of TMS stimulation over bilateral posterior superior temporal sulci. In the pilot study, 13 adults were recruited and underwent 5-day multi-session iTBS over the bilateral pSTS. It revealed significantly immediate effects of iTBS on parent-rate autistic symptoms in adults with ASD. However, no significant changes were observed in the WCST total score and total scores of AQ-self. Moreover, they also found that baseline social-communication symptoms, concurrent psychotropic medication use, and IQ might modulate the effects of iTBS. In their following study, 78 intellectually able children and adolescents with ASD were included. Unfortunately, their results of 4-week blind trial did not support the therapeutic efficacy of this TMS protocol on the clinical symptoms and cognitive performance of social impairment. In this trial, they also carried out a second phase of a 4-week verum TMS stimulation over all the participants included. The results showed that the 8-week active TBS group significantly lowered the total scores of SRS and RBS-R at week 12 in comparison with baseline, while 4-week sham stimulation plus 4-week active stimulation did not. Therefore, they concluded that long-term iTBS is safe and tolerable, which displayed a therapeutic potential to ASD patients. Trials by Ameis et al. were carried out on 16–35-year-old patients with ASD (26). Stimulation over DLPFC for 4 weeks showed that no significant effects on these patients in terms of executive function as compared to sham group. Subjects in the study by Liu et al. received 5 consecutive days of verum or sham iTBS on the rpSTS (27). Those subjects underwent real stimulation did not show significantly larger improvement in emotion recognition than that in the sham group. Of note, functional magnetic resonance imaging (fMRI) data were also acquired in this study, and the results indicated that resting-state functional connectivity (rsFC) between the rpSTS and the left cerebellum significantly decreased by TMS stimulation. This data provided biological evidence for the effects of TMS on the neural activity. Similarly, Moxon-Emre et al. also showed interest in the objective outcome (28). They determined that an excitatory rTMS treatment course could modulate glutamatergic (Glx) levels in emerging adults with ASD and concluded that interventional studies that track glutamatergic markers may provide mechanistic insights into the therapeutic potential of TMS in ASD. Similar to the above tDCS trials, those with moderate and high risk of bias reported significant therapeutic effects of TMS on ASD, despite various stimulation parameters and outcome measurements they applied.

## Discussion

In this systematic review, we summarized and evaluated the existing evidence on the use of NIBS, including tDCS and rTMS, to treat ASD. Unlike the systematic review by Ali Khaleghi et al. who have reviewed the published articles before April 2018, here, twenty-two trials published from 2011 to 2021 were included with 15 (65.2%) published after 2020 (12). However, there is still a very large heterogeneity and variability between studies in terms of patients’ profiles, study designs, stimulation protocols, and outcome measurements. Therefore, so far it is still difficult to draw any conclusions about the promise and therapeutic efficacy of these techniques.

Integral of the success of any clinical trial is the choice of the appropriate patient phenotype and a well-constructed, valid endpoint (46). However, a considerable diversity remains in the existing trials regarding these two issues. Even within those low risk-of-bias trials, different trials chose various outcome measurements as their endpoints, which make it impossible to pool them together to determine the overall effects of NIBS on ASD. To name a few, ATEC was administrated by some trials. The test is a scoring system which consists of four subsets including speech/language/communication (14 items), sociability (20 items), sensory/cognitive awareness (18 items), and health/physical/behavior (25 items) (47). It proves useful to evaluate the effectiveness of various treatments for autistic individuals by researchers, parents, teachers, or caretakers. Other trials also applied scales such as CARS and ABC to evaluate the effectiveness of NIBS on ASD. However, as indicated by Catherine Lord, in the Handbook of Autism and Pervasive Developmental Disorders, these tools were designed to aid in the diagnosis of autism, while as for the measurement of changes in response to treatment, they are not sufficiently sensitive to changes within an individual (48). In addition, some trials may set their endpoint based on the biological function basis of certain brain area of their interests. For instance, based on the role of pSTS in integrating social relevant information to the computational process of the theory of mind, Ni et al. then evaluated the efficacy of TMS on compulsory behavior by repetitive behavior scale-revised (RBS-R) (30). Although more information about the effects of TMS on ASD may be acquired through these exploratory outcome measurements, it may also hinder the parallel comparison across trials, therefore causing a dilemma in reaching clinical consensus.

In addition, heterogeneity also comes from the various ASD symptoms presented in each specific individual. Two individuals could meet DSM or ICD criteria for ASD but present with vastly different behavioral phenotypes as well as variable psychiatric and medical challenges (49). Therefore, it remains challenged to carefully evaluate subject selection and seek to identify more homogeneous subject populations to avoid the possible masking of TMS effects due to intrinsic subject differences. This challenge thus calls for objective brain-based measures such as metabolites alteration, EEG, or fMRI to stratify the population included in the trials. However, a huge gap remains for the translation of any parameters from benchtop to the clinic (50).

With respect to the stimulation protocol including brain area chosen, stimulation intensity, frequency, and times, it varies along with the understanding in the mechanism of ASD pathogenesis. Nonetheless, based on what we have evaluated, TMS over DLPFC may deserve further investigation to confirm its therapeutic effects on the cognitive functions of ASD patients. Although the growing interest in NIBS generated by TMS has led to the revitalization of tDCS, so far, no study has reported different effects of TMS and tDCS. In addition, although NIBS is considered quite safe, even in pediatric populations, one should be cautious about the side effects of any newly designed NIBS protocols (51). Moreover, prior basic research focused more on the temporary effects of NIBS on neural activity and plasticity, there is an urgent need for investigation on the long-term effects and potential structural remodeling effects of this technique. A small number of existing human-involved study have tempted to examine the long-term effects of NIBS on ASD (30). Nonetheless, potential risks should be bear in mind (52). Therefore, more preclinical investigation using mice model or brain organoid to help better understand the mechanism and the effects should be carried out before direct test on human.

In sum, although the heterogeneity impedes us to draw any conclusions about the therapeutic efficacy of NIBS on ASD, we have witnessed an iteration of the existing evidence from reports on exploratory attempts on human to sham-control or even randomized sham-control trials. For the future investigation, to reduce the diversity, a consensus should be reached for choosing reliable assessors to measure the changes in response to the treatment. Objective parameters should be used to stratify the patients included. Furthermore, more basic research on the mechanism and long-term effects of NIBS on brain should be carried out.

## Data availability statement

The original contributions presented in this study are included in the article/Supplementary material, further inquiries can be directed to the corresponding author.

## Author contributions

Both authors conceived the idea of the manuscript and work together to formulate the PICOS search strategy, define the inclusion and exclusion criteria, cross-screen the retrieved items, extract and synthesize the information from the literature, and compose the manuscript.
