# Supplementary material for: Effects of non-invasive neurostimulation on autism spectrum disorder: A systematic review
Source: Front Psychiatry. 2022 Nov 2;13:989905. doi: 10.3389/fpsyt.2022.989905 (PMC9666381; doi:10.3389/fpsyt.2022.989905)
Supplement: Supplementary file 1 [file Table_1.docx]

Supplementary Table 1. Search Strategy for Each Database

| Database | Search strategy |
| --- | --- |
| PubMed | 1. "autism spectrum disorder"[MeSH Terms] OR "autism spectrum disorder"[All Fields] OR asperger*[All Fields] "asperger syndrome"[MeSH Terms] OR "asperger syndrome"[All Fields] OR "autistic disorder"[MeSH Terms] OR "autistic disorder"[All Fields] 2. "transcranial magnetic stimulation"[MeSH Terms] OR "transcranial magnetic stimulation"[All Fields] OR "transcranial direct current stimulation"[MeSH Terms] OR "direct current stimulation"[All Fields] OR "Non-invasive Neurostimulation"[All Fields] OR "transcranial alternating current stimulation"[All Fields]) OR "Theta burst stimulation"[All Fields] OR "Paired associated stimulation"[All Fields] 3. #1 and #2 |
| Embase (1996 to 2021 Week 43） | 1. autism spectrum disorder.mp. OR exp Asperger syndrome/ or Asperger.mp. OR autistic disorder.mp. OR autism.mp. or exp autism/ 2. Non-invasive Neurostimulation.mp. OR Transcranial magnetic stimulation.mp. or exp transcranial magnetic stimulation/ OR transcranial alternating current stimulation.mp. or exp transcranial alternating current stimulation/ OR transcranial direct current stimulation.mp. or exp transcranial direct current stimulation/ OR Theta burst stimulation.mp. OR Paired associated stimulation.mp. 3. #1 and #2 |
| Cochrane library | 1. "autism spectrum disorder"[MeSH Terms] OR "autism spectrum disorder"[All Fields] OR asperger*[All Fields] "asperger syndrome"[MeSH Terms] OR "asperger syndrome"[All Fields] OR "autistic disorder"[MeSH Terms] OR "autistic disorder"[All Fields] OR "ASD"[All Fields] 2. "transcranial magnetic stimulation"[MeSH Terms] OR "transcranial magnetic stimulation"[All Fields] OR "transcranial direct current stimulation"[MeSH Terms] OR "direct current stimulation"[All Fields] OR "Non-invasive Neurostimulation"[All Fields] OR "transcranial alternating current stimulation"[All Fields]) OR "Theta burst stimulation"[All Fields] OR "Paired associated stimulation"[All Fields] (Abbreviations for each methods have also been searched, including "TMS", "DCS", "TACS", "TBS", "PAS") 3. #1 AND #2 |
